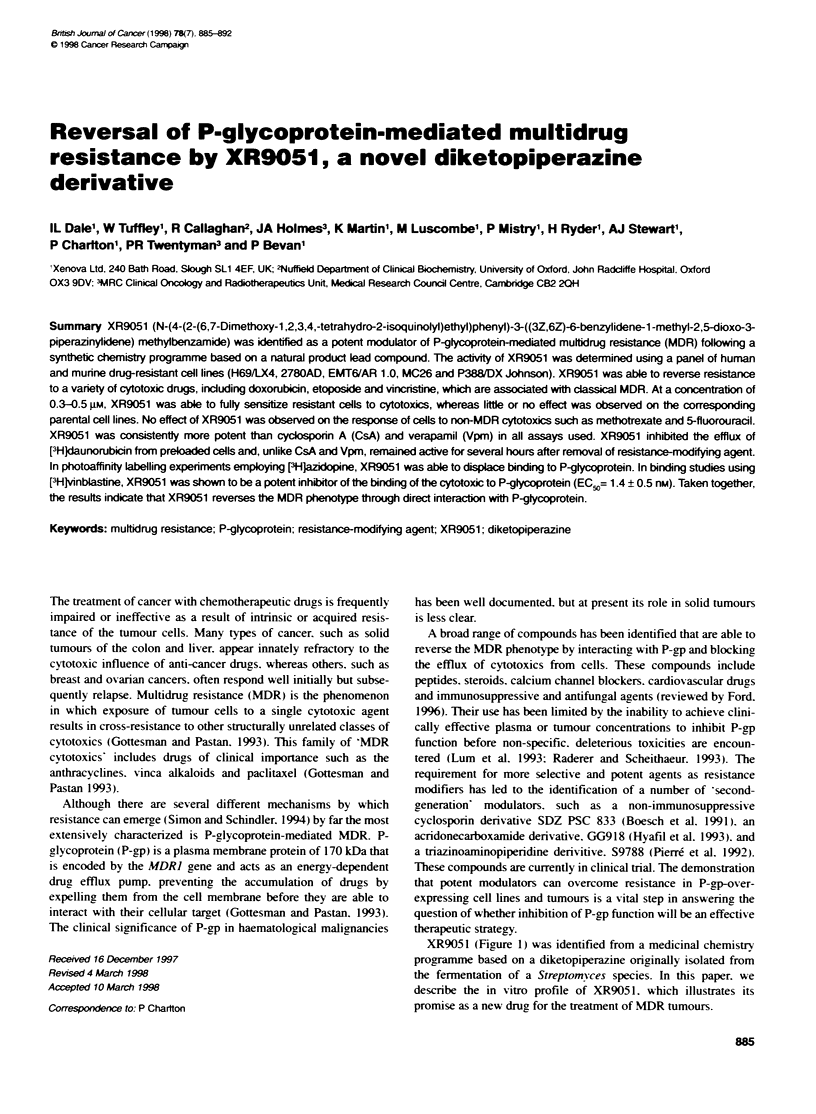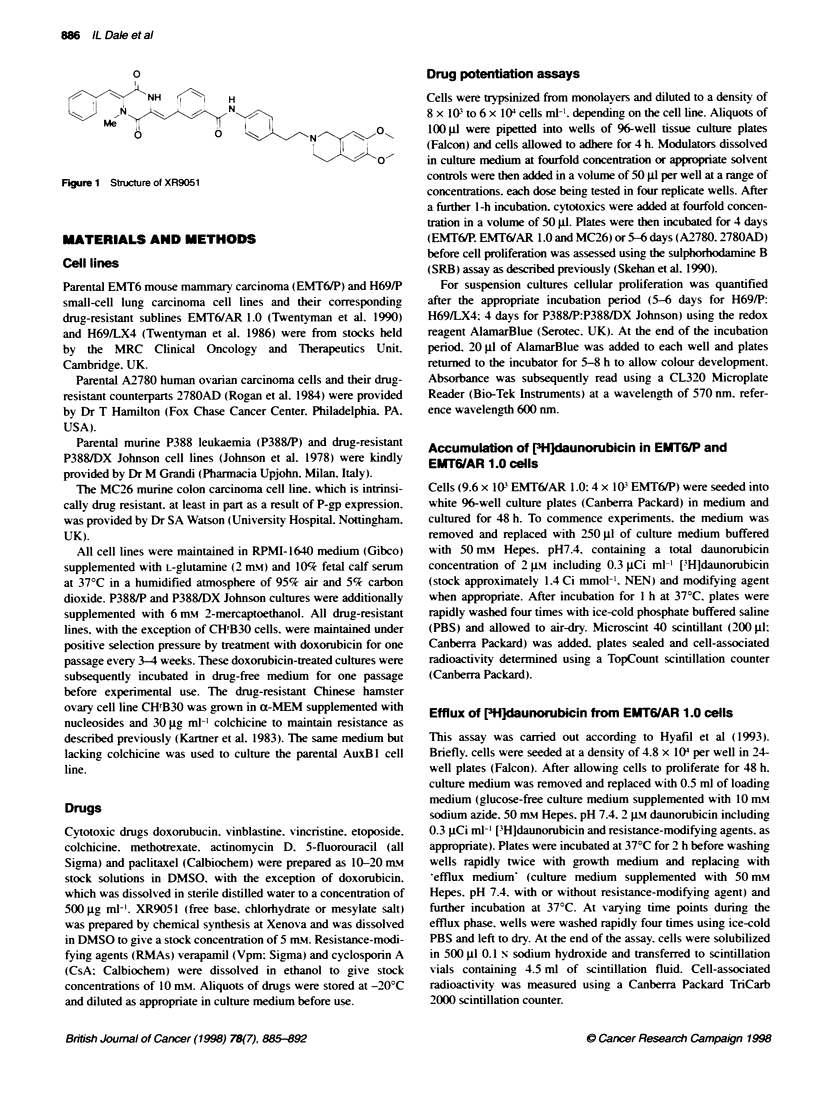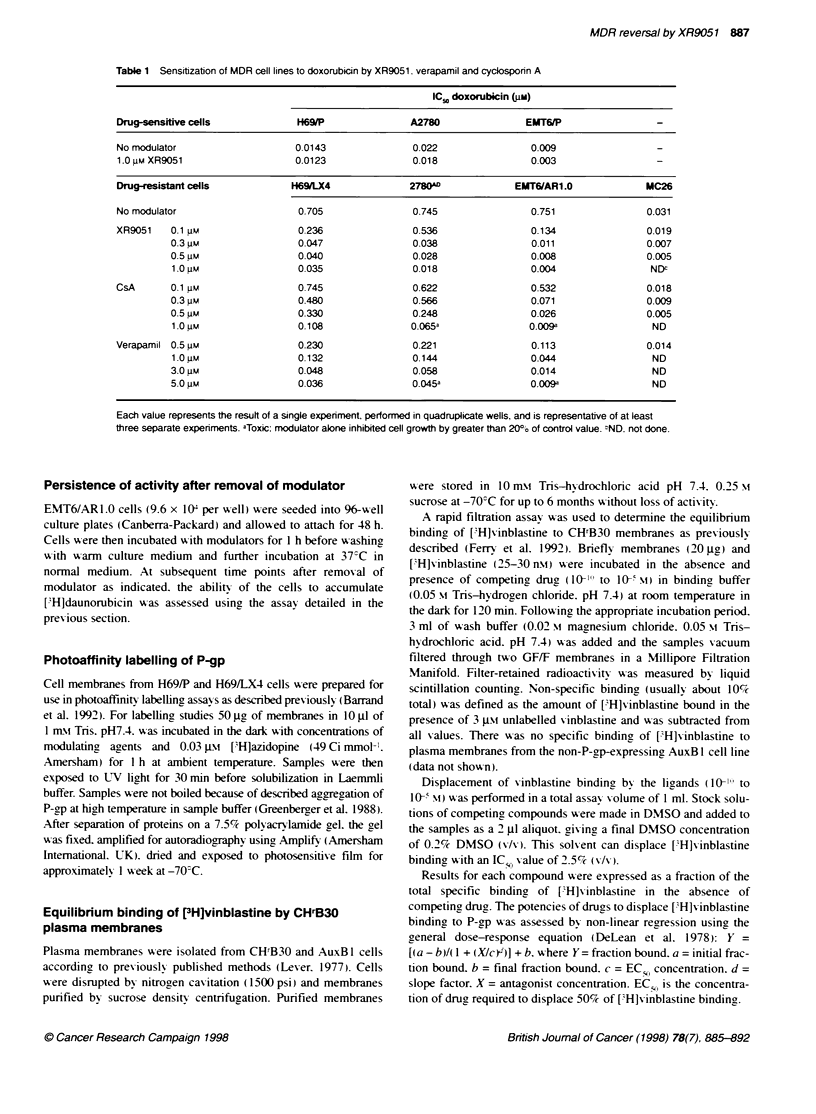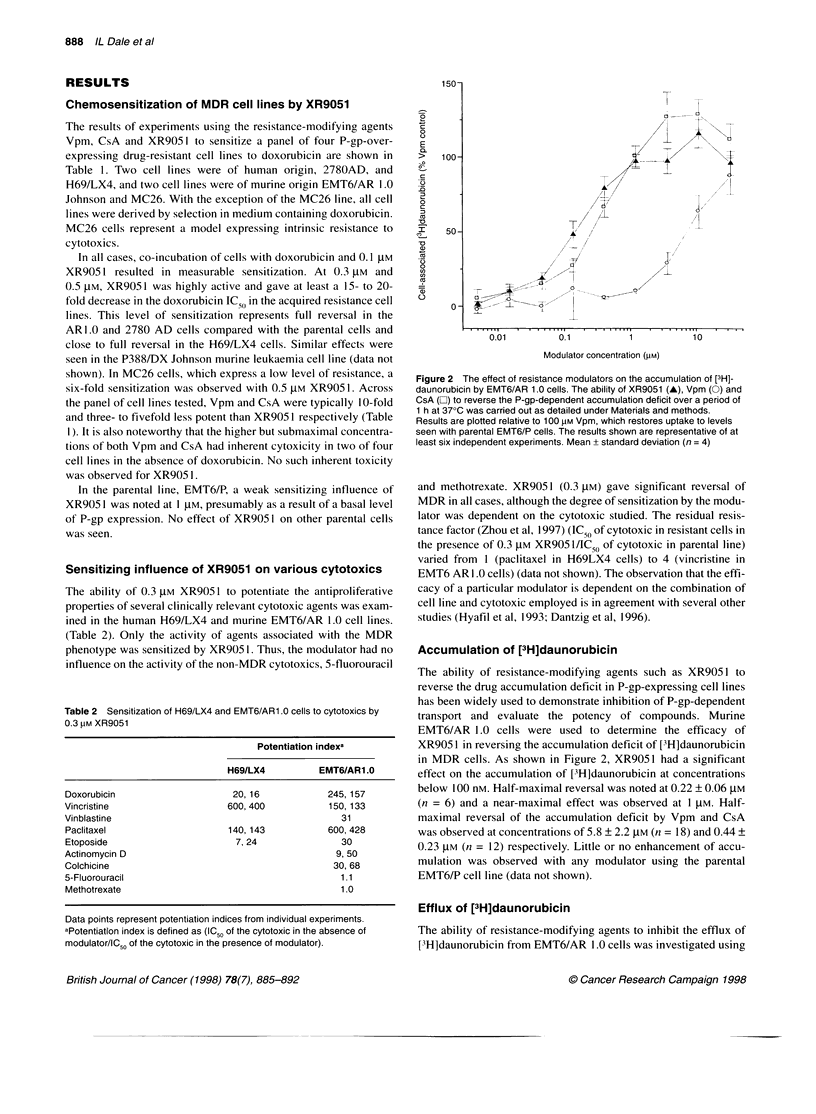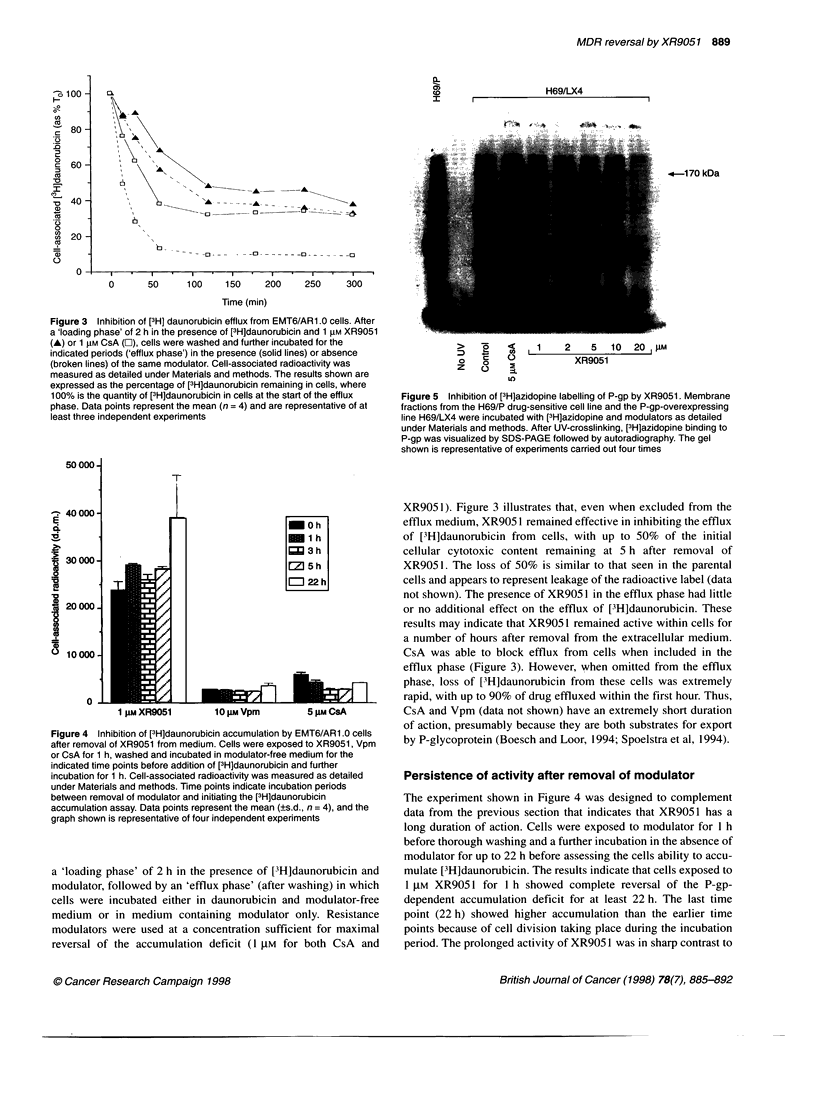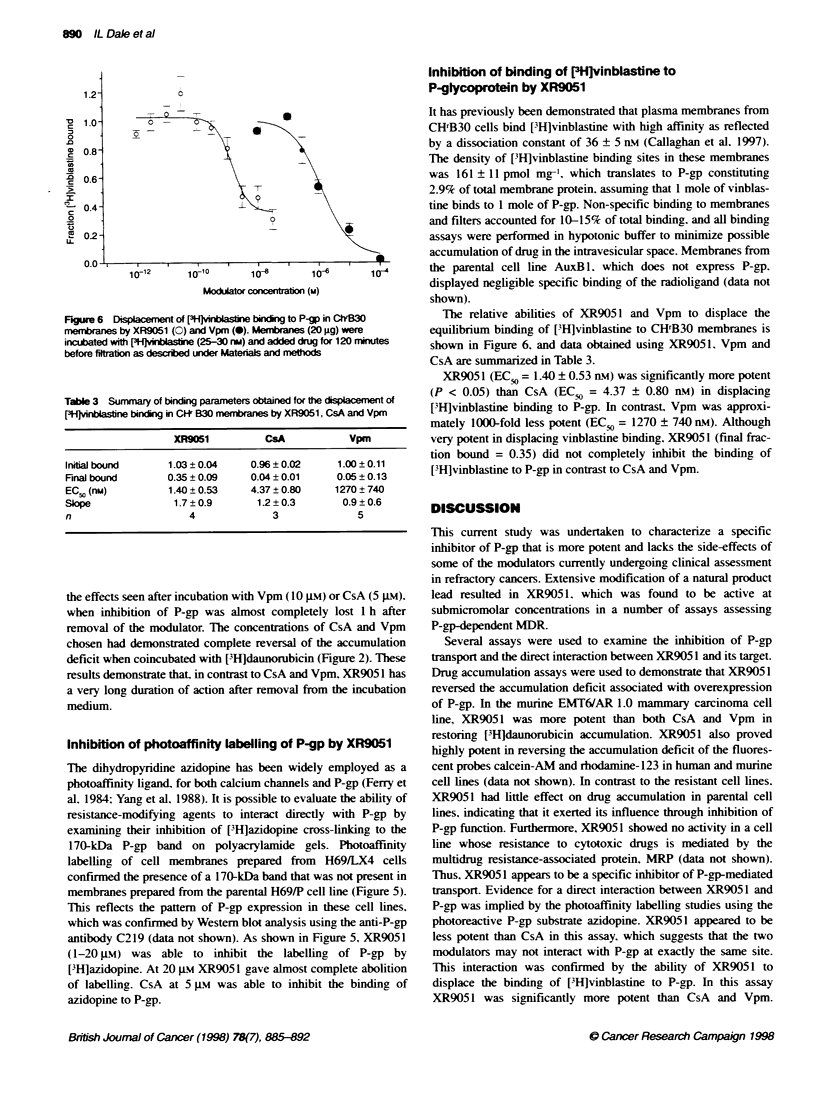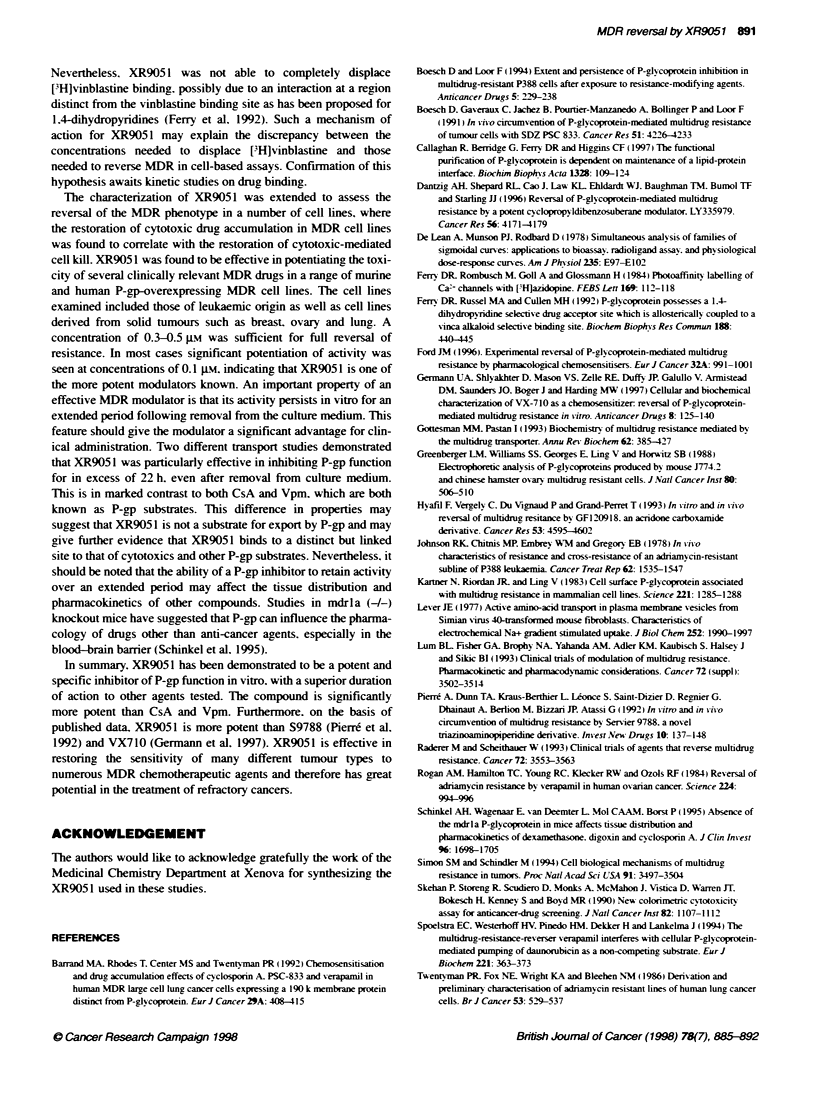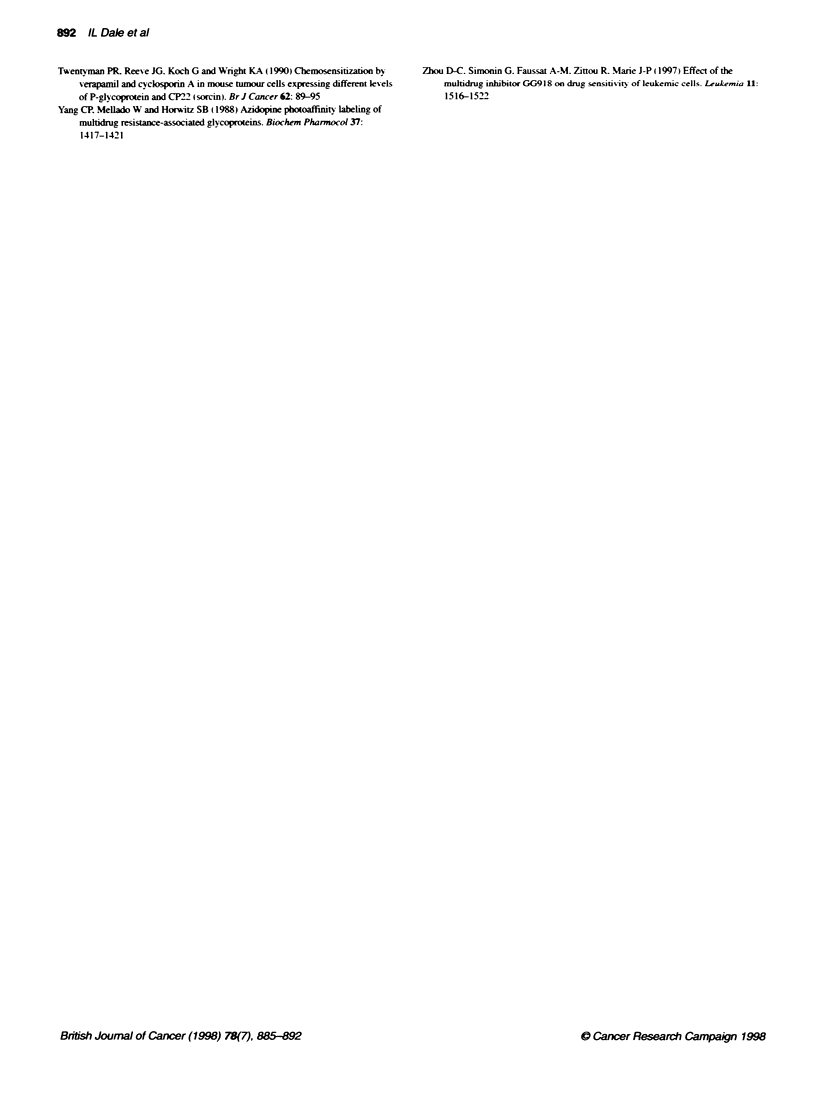# Reversal of P-glycoprotein-mediated multidrug resistance by XR9051, a novel diketopiperazine derivative.

**DOI:** 10.1038/bjc.1998.597

**Published:** 1998-10

**Authors:** I. L. Dale, W. Tuffley, R. Callaghan, J. A. Holmes, K. Martin, M. Luscombe, P. Mistry, H. Ryder, A. J. Stewart, P. Charlton, P. R. Twentyman, P. Bevan

**Affiliations:** Xenova Ltd, Slough, UK.

## Abstract

**Images:**


British Journal of Cancer (1998) 78(7), 885–892
© 1998 Cancer Research Campaign

### Reversal of P-glycoprotein-mediated multidrug resistance by XR9051, a novel diketopiperazine derivative

IL Dale[1], W Tuffley[1], R Callaghan[2], JA Holmes[3], K Martin[1], M Luscombe[1], P Mistry[1], H Ryder[1], AJ Stewart[1], P Charlton[1], PR Twentyman[3] and P Bevan[1]

[1]Xenova Ltd, 240 Bath Road, Slough SL1 4EF, UK; [2]Nuffield Department of Clinical Biochemistry, University of Oxford, John Radcliffe Hospital, Oxford OX3 9DV; [3]MRC Clinical Oncology and Radiotherapeutics Unit, Medical Research Council Centre, Cambridge CB2 2QH

**Summary** XR9051 (N-(4-(2-(6,7-Dimethoxy-1,2,3,4,-tetrahydro-2-isoquinolyl)ethyl)phenyl)-3-((3Z,6Z)-6-benzylidene-1-methyl-2,5-dioxo-3-piperazinylidene) methylbenzamide) was identified as a potent modulator of P-glycoprotein-mediated multidrug resistance (MDR) following a synthetic chemistry programme based on a natural product lead compound. The activity of XR9051 was determined using a panel of human and murine drug-resistant cell lines (H69/LX4, 2780AD, EMT6/AR 1.0, MC26 and P388/DX Johnson). XR9051 was able to reverse resistance to a variety of cytotoxic drugs, including doxorubicin, etoposide and vincristine, which are associated with classical MDR. At a concentration of 0.3–0.5 μM, XR9051 was able to fully sensitize resistant cells to cytotoxics, whereas little or no effect was observed on the corresponding parental cell lines. No effect of XR9051 was observed on the response of cells to non-MDR cytotoxics such as methotrexate and 5-fluorouracil. XR9051 was consistently more potent than cyclosporin A (CsA) and verapamil (Vpm) in all assays used. XR9051 inhibited the efflux of [$^3$H]daunorubicin from preloaded cells and, unlike CsA and Vpm, remained active for several hours after removal of resistance-modifying agent. In photoaffinity labelling experiments employing [$^3$H]azidopine, XR9051 was able to displace binding to P-glycoprotein. In binding studies using [$^3$H]vinblastine, XR9051 was shown to be a potent inhibitor of the binding of the cytotoxic to P-glycoprotein ($EC_{50}$ = 1.4 ± 0.5 nM). Taken together, the results indicate that XR9051 reverses the MDR phenotype through direct interaction with P-glycoprotein.

**Keywords:** multidrug resistance; P-glycoprotein; resistance-modifying agent; XR9051; diketopiperazine

The treatment of cancer with chemotherapeutic drugs is frequently impaired or ineffective as a result of intrinsic or acquired resistance of the tumour cells. Many types of cancer, such as solid tumours of the colon and liver, appear innately refractory to the cytotoxic influence of anti-cancer drugs, whereas others, such as breast and ovarian cancers, often respond well initially but subsequently relapse. Multidrug resistance (MDR) is the phenomenon in which exposure of tumour cells to a single cytotoxic agent results in cross-resistance to other structurally unrelated classes of cytotoxics (Gottesman and Pastan, 1993). This family of 'MDR cytotoxics' includes drugs of clinical importance such as the anthracyclines, vinca alkaloids and paclitaxel (Gottesman and Pastan 1993).

Although there are several different mechanisms by which resistance can emerge (Simon and Schindler, 1994) by far the most extensively characterized is P-glycoprotein-mediated MDR. P-glycoprotein (P-gp) is a plasma membrane protein of 170 kDa that is encoded by the *MDR1* gene and acts as an energy-dependent drug efflux pump, preventing the accumulation of drugs by expelling them from the cell membrane before they are able to interact with their cellular target (Gottesman and Pastan, 1993). The clinical significance of P-gp in haematological malignancies has been well documented, but at present its role in solid tumours is less clear.

A broad range of compounds has been identified that are able to reverse the MDR phenotype by interacting with P-gp and blocking the efflux of cytotoxics from cells. These compounds include peptides, steroids, calcium channel blockers, cardiovascular drugs and immunosuppressive and antifungal agents (reviewed by Ford, 1996). Their use has been limited by the inability to achieve clinically effective plasma or tumour concentrations to inhibit P-gp function before non-specific, deleterious toxicities are encountered (Lum et al, 1993; Raderer and Scheithaeur, 1993). The requirement for more selective and potent agents as resistance modifiers has led to the identification of a number of 'second-generation' modulators, such as a non-immunosuppressive cyclosporin derivative SDZ PSC 833 (Boesch et al, 1991), an acridonecarboxamide derivative, GG918 (Hyafil et al, 1993), and a triazinoaminopiperidine derivitive, S9788 (Pierré et al, 1992). These compounds are currently in clinical trial. The demonstration that potent modulators can overcome resistance in P-gp-over-expressing cell lines and tumours is a vital step in answering the question of whether inhibition of P-gp function will be an effective therapeutic strategy.

XR9051 (Figure 1) was identified from a medicinal chemistry programme based on a diketopiperazine originally isolated from the fermentation of a *Streptomyces* species. In this paper, we describe the in vitro profile of XR9051, which illustrates its promise as a new drug for the treatment of MDR tumours.

*Received 16 December 1997*
*Revised 4 March 1998*
*Accepted 10 March 1998*

*Correspondence to:* P Charlton

**Figure 1** Structure of XR9051

### MATERIALS AND METHODS

### Cell lines

Parental EMT6 mouse mammary carcinoma (EMT6/P) and H69/P small-cell lung carcinoma cell lines and their corresponding drug-resistant sublines EMT6/AR 1.0 (Twentyman et al. 1990) and H69/LX4 (Twentyman et al. 1986) were from stocks held by the MRC Clinical Oncology and Therapeutics Unit, Cambridge, UK.

Parental A2780 human ovarian carcinoma cells and their drug-resistant counterparts 2780AD (Rogan et al. 1984) were provided by Dr T Hamilton (Fox Chase Cancer Center, Philadelphia, PA, USA).

Parental murine P388 leukaemia (P388/P) and drug-resistant P388/DX Johnson cell lines (Johnson et al. 1978) were kindly provided by Dr M Grandi (Pharmacia Upjohn, Milan, Italy).

The MC26 murine colon carcinoma cell line, which is intrinsically drug resistant, at least in part as a result of P-gp expression, was provided by Dr SA Watson (University Hospital, Nottingham, UK).

All cell lines were maintained in RPMI-1640 medium (Gibco) supplemented with L-glutamine (2 mM) and 10% fetal calf serum at 37°C in a humidified atmosphere of 95% air and 5% carbon dioxide. P388/P and P388/DX Johnson cultures were additionally supplemented with 6 mM 2-mercaptoethanol. All drug-resistant lines, with the exception of $CH^rB30$ cells, were maintained under positive selection pressure by treatment with doxorubicin for one passage every 3–4 weeks. These doxorubicin-treated cultures were subsequently incubated in drug-free medium for one passage before experimental use. The drug-resistant Chinese hamster ovary cell line $CH^rB30$ was grown in α-MEM supplemented with nucleosides and 30 μg $ml^{-1}$ colchicine to maintain resistance as described previously (Kartner et al. 1983). The same medium but lacking colchicine was used to culture the parental AuxB1 cell line.

### Drugs

Cytotoxic drugs doxorubucin, vinblastine, vincristine, etoposide, colchicine, methotrexate, actinomycin D, 5-fluorouracil (all Sigma) and paclitaxel (Calbiochem) were prepared as 10–20 mM stock solutions in DMSO, with the exception of doxorubicin, which was dissolved in sterile distilled water to a concentration of 500 μg $ml^{-1}$. XR9051 (free base, chlorhydrate or mesylate salt) was prepared by chemical synthesis at Xenova and was dissolved in DMSO to give a stock concentration of 5 mM. Resistance-modifying agents (RMAs) verapamil (Vpm; Sigma) and cyclosporin A (CsA; Calbiochem) were dissolved in ethanol to give stock concentrations of 10 mM. Aliquots of drugs were stored at −20°C and diluted as appropriate in culture medium before use.

### Drug potentiation assays

Cells were trypsinized from monolayers and diluted to a density of $8 \times 10^3$ to $6 \times 10^4$ cells $ml^{-1}$, depending on the cell line. Aliquots of 100 μl were pipetted into wells of 96-well tissue culture plates (Falcon) and cells allowed to adhere for 4 h. Modulators dissolved in culture medium at fourfold concentration or appropriate solvent controls were then added in a volume of 50 μl per well at a range of concentrations, each dose being tested in four replicate wells. After a further 1-h incubation, cytotoxics were added at fourfold concentration in a volume of 50 μl. Plates were then incubated for 4 days (EMT6/P, EMT6/AR 1.0 and MC26) or 5–6 days (A2780, 2780AD) before cell proliferation was assessed using the sulphorhodamine B (SRB) assay as described previously (Skehan et al. 1990).

For suspension cultures cellular proliferation was quantified after the appropriate incubation period (5–6 days for H69/P: H69/LX4; 4 days for P388/P:P388/DX Johnson) using the redox reagent AlamarBlue (Serotec, UK). At the end of the incubation period, 20 μl of AlamarBlue was added to each well and plates returned to the incubator for 5–8 h to allow colour development. Absorbance was subsequently read using a CL320 Microplate Reader (Bio-Tek Instruments) at a wavelength of 570 nm, reference wavelength 600 nm.

### Accumulation of [$^3$H]daunorubicin in EMT6/P and EMT6/AR 1.0 cells

Cells ($9.6 \times 10^3$ EMT6/AR 1.0; $4 \times 10^3$ EMT6/P) were seeded into white 96-well culture plates (Canberra Packard) in medium and cultured for 48 h. To commence experiments, the medium was removed and replaced with 250 μl of culture medium buffered with 50 mM Hepes, pH7.4, containing a total daunorubicin concentration of 2 μM including 0.3 μCi $ml^{-1}$ [$^3$H]daunorubicin (stock approximately 1.4 Ci $mmol^{-1}$, NEN) and modifying agent when appropriate. After incubation for 1 h at 37°C, plates were rapidly washed four times with ice-cold phosphate buffered saline (PBS) and allowed to air-dry. Microscint 40 scintillant (200 μl; Canberra Packard) was added, plates sealed and cell-associated radioactivity determined using a TopCount scintillation counter (Canberra Packard).

### Efflux of [$^3$H]daunorubicin from EMT6/AR 1.0 cells

This assay was carried out according to Hyafil et al (1993). Briefly, cells were seeded at a density of $4.8 \times 10^4$ per well in 24-well plates (Falcon). After allowing cells to proliferate for 48 h, culture medium was removed and replaced with 0.5 ml of loading medium (glucose-free culture medium supplemented with 10 mM sodium azide, 50 mM Hepes, pH 7.4, 2 μM daunorubicin including 0.3 μCi $ml^{-1}$ [$^3$H]daunorubicin and resistance-modifying agents, as appropriate). Plates were incubated at 37°C for 2 h before washing wells rapidly twice with growth medium and replacing with 'efflux medium' (culture medium supplemented with 50 mM Hepes, pH 7.4, with or without resistance-modifying agent) and further incubation at 37°C. At varying time points during the efflux phase, wells were washed rapidly four times using ice-cold PBS and left to dry. At the end of the assay, cells were solubilized in 500 μl 0.1 N sodium hydroxide and transferred to scintillation vials containing 4.5 ml of scintillation fluid. Cell-associated radioactivity was measured using a Canberra Packard TriCarb 2000 scintillation counter.

© Cancer Research Campaign 1998

**Table 1** Sensitization of MDR cell lines to doxorubicin by XR9051, verapamil and cyclosporin A

| | | $IC_{50}$ doxorubicin (μM) | | | |
|---|---|---|---|---|---|
| **Drug-sensitive cells** | | **H69/P** | **A2780** | **EMT6/P** | – |
| No modulator | | 0.0143 | 0.022 | 0.009 | – |
| 1.0 μM XR9051 | | 0.0123 | 0.018 | 0.003 | – |
| **Drug-resistant cells** | | **H69/LX4** | **2780[AD]** | **EMT6/AR1.0** | **MC26** |
| No modulator | | 0.705 | 0.745 | 0.751 | 0.031 |
| XR9051 | 0.1 μM | 0.236 | 0.536 | 0.134 | 0.019 |
| | 0.3 μM | 0.047 | 0.038 | 0.011 | 0.007 |
| | 0.5 μM | 0.040 | 0.028 | 0.008 | 0.005 |
| | 1.0 μM | 0.035 | 0.018 | 0.004 | ND[c] |
| CsA | 0.1 μM | 0.745 | 0.622 | 0.532 | 0.018 |
| | 0.3 μM | 0.480 | 0.566 | 0.071 | 0.009 |
| | 0.5 μM | 0.330 | 0.248 | 0.026 | 0.005 |
| | 1.0 μM | 0.108 | 0.065[a] | 0.009[a] | ND |
| Verapamil | 0.5 μM | 0.230 | 0.221 | 0.113 | 0.014 |
| | 1.0 μM | 0.132 | 0.144 | 0.044 | ND |
| | 3.0 μM | 0.048 | 0.058 | 0.014 | ND |
| | 5.0 μM | 0.036 | 0.045[a] | 0.009[a] | ND |

Each value represents the result of a single experiment, performed in quadruplicate wells, and is representative of at least three separate experiments. [a]Toxic: modulator alone inhibited cell growth by greater than 20% of control value. [c]ND, not done.

### Persistence of activity after removal of modulator

EMT6/AR1.0 cells ($9.6 \times 10^4$ per well) were seeded into 96-well culture plates (Canberra-Packard) and allowed to attach for 48 h. Cells were then incubated with modulators for 1 h before washing with warm culture medium and further incubation at 37°C in normal medium. At subsequent time points after removal of modulator as indicated, the ability of the cells to accumulate [$^3$H]daunorubicin was assessed using the assay detailed in the previous section.

### Photoaffinity labelling of P-gp

Cell membranes from H69/P and H69/LX4 cells were prepared for use in photoaffinity labelling assays as described previously (Barrand et al, 1992). For labelling studies 50 μg of membranes in 10 μl of 1 mM Tris, pH7.4, was incubated in the dark with concentrations of modulating agents and 0.03 μM [$^3$H]azidopine (49 Ci mmol$^{-1}$, Amersham) for 1 h at ambient temperature. Samples were then exposed to UV light for 30 min before solubilization in Laemmli buffer. Samples were not boiled because of described aggregation of P-gp at high temperature in sample buffer (Greenberger et al, 1988). After separation of proteins on a 7.5% polyacrylamide gel, the gel was fixed, amplified for autoradiography using Amplify (Amersham International, UK), dried and exposed to photosensitive film for approximately 1 week at –70°C.

### Equilibrium binding of [$^3$H]vinblastine by CH$^r$B30 plasma membranes

Plasma membranes were isolated from CH$^r$B30 and AuxB1 cells according to previously published methods (Lever, 1977). Cells were disrupted by nitrogen cavitation (1500 psi) and membranes purified by sucrose density centrifugation. Purified membranes were stored in 10 mM Tris–hydrochloric acid pH 7.4, 0.25 M sucrose at –70°C for up to 6 months without loss of activity.

A rapid filtration assay was used to determine the equilibrium binding of [$^3$H]vinblastine to CH$^r$B30 membranes as previously described (Ferry et al, 1992). Briefly membranes (20 μg) and [$^3$H]vinblastine (25–30 nM) were incubated in the absence and presence of competing drug ($10^{-10}$ to $10^{-5}$ M) in binding buffer (0.05 M Tris–hydrogen chloride, pH 7.4) at room temperature in the dark for 120 min. Following the appropriate incubation period, 3 ml of wash buffer (0.02 M magnesium chloride, 0.05 M Tris–hydrochloric acid, pH 7.4) was added and the samples vacuum filtered through two GF/F membranes in a Millipore Filtration Manifold. Filter-retained radioactivity was measured by liquid scintillation counting. Non-specific binding (usually about 10% total) was defined as the amount of [$^3$H]vinblastine bound in the presence of 3 μM unlabelled vinblastine and was subtracted from all values. There was no specific binding of [$^3$H]vinblastine to plasma membranes from the non-P-gp-expressing AuxB1 cell line (data not shown).

Displacement of vinblastine binding by the ligands ($10^{-10}$ to $10^{-5}$ M) was performed in a total assay volume of 1 ml. Stock solutions of competing compounds were made in DMSO and added to the samples as a 2 μl aliquot, giving a final DMSO concentration of 0.2% DMSO (v/v). This solvent can displace [$^3$H]vinblastine binding with an $IC_{50}$ value of 2.5% (v/v).

Results for each compound were expressed as a fraction of the total specific binding of [$^3$H]vinblastine in the absence of competing drug. The potencies of drugs to displace [$^3$H]vinblastine binding to P-gp was assessed by non-linear regression using the general dose–response equation (DeLean et al, 1978): $Y = [(a - b)/(1 + (X/c)^d)] + b$, where $Y$ = fraction bound, $a$ = initial fraction bound, $b$ = final fraction bound, $c$ = $EC_{50}$ concentration, $d$ = slope factor, $X$ = antagonist concentration. $EC_{50}$ is the concentration of drug required to displace 50% of [$^3$H]vinblastine binding.

© Cancer Research Campaign 1998 

### RESULTS

### Chemosensitization of MDR cell lines by XR9051

The results of experiments using the resistance-modifying agents Vpm, CsA and XR9051 to sensitize a panel of four P-gp-overexpressing drug-resistant cell lines to doxorubicin are shown in Table 1. Two cell lines were of human origin, 2780AD, and H69/LX4, and two cell lines were of murine origin EMT6/AR 1.0 Johnson and MC26. With the exception of the MC26 line, all cell lines were derived by selection in medium containing doxorubicin. MC26 cells represent a model expressing intrinsic resistance to cytotoxics.

In all cases, co-incubation of cells with doxorubicin and 0.1 μM XR9051 resulted in measurable sensitization. At 0.3 μM and 0.5 μM, XR9051 was highly active and gave at least a 15- to 20-fold decrease in the doxorubicin $IC_{50}$ in the acquired resistance cell lines. This level of sensitization represents full reversal in the AR1.0 and 2780 AD cells compared with the parental cells and close to full reversal in the H69/LX4 cells. Similar effects were seen in the P388/DX Johnson murine leukaemia cell line (data not shown). In MC26 cells, which express a low level of resistance, a six-fold sensitization was observed with 0.5 μM XR9051. Across the panel of cell lines tested, Vpm and CsA were typically 10-fold and three- to fivefold less potent than XR9051 respectively (Table 1). It is also noteworthy that the higher but submaximal concentrations of both Vpm and CsA had inherent cytoxicity in two of four cell lines in the absence of doxorubicin. No such inherent toxicity was observed for XR9051.

In the parental line, EMT6/P, a weak sensitizing influence of XR9051 was noted at 1 μM, presumably as a result of a basal level of P-gp expression. No effect of XR9051 on other parental cells was seen.

### Sensitizing influence of XR9051 on various cytotoxics

The ability of 0.3 μM XR9051 to potentiate the antiproliferative properties of several clinically relevant cytotoxic agents was examined in the human H69/LX4 and murine EMT6/AR 1.0 cell lines. (Table 2). Only the activity of agents associated with the MDR phenotype was sensitized by XR9051. Thus, the modulator had no influence on the activity of the non-MDR cytotoxics, 5-fluorouracil and methotrexate. XR9051 (0.3 μM) gave significant reversal of MDR in all cases, although the degree of sensitization by the modulator was dependent on the cytotoxic studied. The residual resistance factor (Zhou et al, 1997) ($IC_{50}$ of cytotoxic in resistant cells in the presence of 0.3 μM XR9051/$IC_{50}$ of cytotoxic in parental line) varied from 1 (paclitaxel in H69LX4 cells) to 4 (vincristine in EMT6 AR1.0 cells) (data not shown). The observation that the efficacy of a particular modulator is dependent on the combination of cell line and cytotoxic employed is in agreement with several other studies (Hyafil et al, 1993; Dantzig et al, 1996).

**Table 2** Sensitization of H69/LX4 and EMT6/AR1.0 cells to cytotoxics by 0.3 μM XR9051

| | Potentiation index[a] | |
|---|---|---|
| | H69/LX4 | EMT6/AR1.0 |
| Doxorubicin | 20, 16 | 245, 157 |
| Vincristine | 600, 400 | 150, 133 |
| Vinblastine | | 31 |
| Paclitaxel | 140, 143 | 600, 428 |
| Etoposide | 7, 24 | 30 |
| Actinomycin D | | 9, 50 |
| Colchicine | | 30, 68 |
| 5-Fluorouracil | | 1.1 |
| Methotrexate | | 1.0 |

Data points represent potentiation indices from individual experiments. [a]Potentiation index is defined as ($IC_{50}$ of the cytotoxic in the absence of modulator/$IC_{50}$ of the cytotoxic in the presence of modulator).

**Figure 2** The effect of resistance modulators on the accumulation of [$^3$H]-daunorubicin by EMT6/AR 1.0 cells. The ability of XR9051 (▲), Vpm (○) and CsA (□) to reverse the P-gp-dependent accumulation deficit over a period of 1 h at 37°C was carried out as detailed under Materials and methods. Results are plotted relative to 100 μM Vpm, which restores uptake to levels seen with parental EMT6/P cells. The results shown are representative of at least six independent experiments. Mean ± standard deviation ($n$ = 4)

### Accumulation of [$^3$H]daunorubicin

The ability of resistance-modifying agents such as XR9051 to reverse the drug accumulation deficit in P-gp-expressing cell lines has been widely used to demonstrate inhibition of P-gp-dependent transport and evaluate the potency of compounds. Murine EMT6/AR 1.0 cells were used to determine the efficacy of XR9051 in reversing the accumulation deficit of [$^3$H]daunorubicin in MDR cells. As shown in Figure 2, XR9051 had a significant effect on the accumulation of [$^3$H]daunorubicin at concentrations below 100 nM. Half-maximal reversal was noted at 0.22 ± 0.06 μM ($n$ = 6) and a near-maximal effect was observed at 1 μM. Half-maximal reversal of the accumulation deficit by Vpm and CsA was observed at concentrations of 5.8 ± 2.2 μM ($n$ = 18) and 0.44 ± 0.23 μM ($n$ = 12) respectively. Little or no enhancement of accumulation was observed with any modulator using the parental EMT6/P cell line (data not shown).

### Efflux of [$^3$H]daunorubicin

The ability of resistance-modifying agents to inhibit the efflux of [$^3$H]daunorubicin from EMT6/AR 1.0 cells was investigated using

© Cancer Research Campaign 1998

**Figure 3** Inhibition of [$^3$H] daunorubicin efflux from EMT6/AR1.0 cells. After a 'loading phase' of 2 h in the presence of [$^3$H]daunorubicin and 1 μM XR9051 (▲) or 1 μM CsA (□), cells were washed and further incubated for the indicated periods ('efflux phase') in the presence (solid lines) or absence (broken lines) of the same modulator. Cell-associated radioactivity was measured as detailed under Materials and methods. The results shown are expressed as the percentage of [$^3$H]daunorubicin remaining in cells, where 100% is the quantity of [$^3$H]daunorubicin in cells at the start of the efflux phase. Data points represent the mean ($n$ = 4) and are representative of at least three independent experiments

**Figure 4** Inhibition of [$^3$H]daunorubicin accumulation by EMT6/AR1.0 cells after removal of XR9051 from medium. Cells were exposed to XR9051, Vpm or CsA for 1 h, washed and incubated in modulator-free medium for the indicated time points before addition of [$^3$H]daunorubicin and further incubation for 1 h. Cell-associated radioactivity was measured as detailed under Materials and methods. Time points indicate incubation periods between removal of modulator and initiating the [$^3$H]daunorubicin accumulation assay. Data points represent the mean (±s.d., $n$ = 4), and the graph shown is representative of four independent experiments

**Figure 5** Inhibition of [$^3$H]azidopine labelling of P-gp by XR9051. Membrane fractions from the H69/P drug-sensitive cell line and the P-gp-overexpressing line H69/LX4 were incubated with [$^3$H]azidopine and modulators as detailed under Materials and methods. After UV-crosslinking, [$^3$H]azidopine binding to P-gp was visualized by SDS-PAGE followed by autoradiography. The gel shown is representative of experiments carried out four times a 'loading phase' of 2 h in the presence of [$^3$H]daunorubicin and modulator, followed by an 'efflux phase' (after washing) in which cells were incubated either in daunorubicin and modulator-free medium or in medium containing modulator only. Resistance modulators were used at a concentration sufficient for maximal reversal of the accumulation deficit (1 μM for both CsA and XR9051). Figure 3 illustrates that, even when excluded from the efflux medium, XR9051 remained effective in inhibiting the efflux of [$^3$H]daunorubicin from cells, with up to 50% of the initial cellular cytotoxic content remaining at 5 h after removal of XR9051. The loss of 50% is similar to that seen in the parental cells and appears to represent leakage of the radioactive label (data not shown). The presence of XR9051 in the efflux phase had little or no additional effect on the efflux of [$^3$H]daunorubicin. These results may indicate that XR9051 remained active within cells for a number of hours after removal from the extracellular medium. CsA was able to block efflux from cells when included in the efflux phase (Figure 3). However, when omitted from the efflux phase, loss of [$^3$H]daunorubicin from these cells was extremely rapid, with up to 90% of drug effluxed within the first hour. Thus, CsA and Vpm (data not shown) have an extremely short duration of action, presumably because they are both substrates for export by P-glycoprotein (Boesch and Loor, 1994; Spoelstra et al, 1994).

### Persistence of activity after removal of modulator

The experiment shown in Figure 4 was designed to complement data from the previous section that indicates that XR9051 has a long duration of action. Cells were exposed to modulator for 1 h before thorough washing and a further incubation in the absence of modulator for up to 22 h before assessing the cells ability to accumulate [$^3$H]daunorubicin. The results indicate that cells exposed to 1 μM XR9051 for 1 h showed complete reversal of the P-gp-dependent accumulation deficit for at least 22 h. The last time point (22 h) showed higher accumulation than the earlier time points because of cell division taking place during the incubation period. The prolonged activity of XR9051 was in sharp contrast to

© Cancer Research Campaign 1998

**Figure 6** Displacement of [$^3$H]vinblastine binding to P-gp in CH$^r$B30 membranes by XR9051 (○) and Vpm (●). Membranes (20 μg) were incubated with [$^3$H]vinblastine (25–30 nM) and added drug for 120 minutes before filtration as described under Materials and methods

**Table 3** Summary of binding parameters obtained for the displacement of [$^3$H]vinblastine binding in CH$^r$ B30 membranes by XR9051, CsA and Vpm

| | XR9051 | CsA | Vpm |
|---|---|---|---|
| Initial bound | 1.03 ± 0.04 | 0.96 ± 0.02 | 1.00 ± 0.11 |
| Final bound | 0.35 ± 0.09 | 0.04 ± 0.01 | 0.05 ± 0.13 |
| $EC_{50}$ (nM) | 1.40 ± 0.53 | 4.37 ± 0.80 | 1270 ± 740 |
| Slope | 1.7 ± 0.9 | 1.2 ± 0.3 | 0.9 ± 0.6 |
| *n* | 4 | 3 | 5 |

the effects seen after incubation with Vpm (10 μM) or CsA (5 μM), when inhibition of P-gp was almost completely lost 1 h after removal of the modulator. The concentrations of CsA and Vpm chosen had demonstrated complete reversal of the accumulation deficit when coincubated with [$^3$H]daunorubicin (Figure 2). These results demonstrate that, in contrast to CsA and Vpm, XR9051 has a very long duration of action after removal from the incubation medium.

### Inhibition of photoaffinity labelling of P-gp by XR9051

The dihydropyridine azidopine has been widely employed as a photoaffinity ligand, for both calcium channels and P-gp (Ferry et al, 1984; Yang et al, 1988). It is possible to evaluate the ability of resistance-modifying agents to interact directly with P-gp by examining their inhibition of [$^3$H]azidopine cross-linking to the 170-kDa P-gp band on polyacrylamide gels. Photoaffinity labelling of cell membranes prepared from H69/LX4 cells confirmed the presence of a 170-kDa band that was not present in membranes prepared from the parental H69/P cell line (Figure 5). This reflects the pattern of P-gp expression in these cell lines, which was confirmed by Western blot analysis using the anti-P-gp antibody C219 (data not shown). As shown in Figure 5, XR9051 (1–20 μM) was able to inhibit the labelling of P-gp by [$^3$H]azidopine. At 20 μM XR9051 gave almost complete abolition of labelling. CsA at 5 μM was able to inhibit the binding of azidopine to P-gp.

### Inhibition of binding of [$^3$H]vinblastine to P-glycoprotein by XR9051

It has previously been demonstrated that plasma membranes from CH$^r$B30 cells bind [$^3$H]vinblastine with high affinity as reflected by a dissociation constant of 36 ± 5 nM (Callaghan et al, 1997). The density of [$^3$H]vinblastine binding sites in these membranes was 161 ± 11 pmol $mg^{-1}$, which translates to P-gp constituting 2.9% of total membrane protein, assuming that 1 mole of vinblastine binds to 1 mole of P-gp. Non-specific binding to membranes and filters accounted for 10–15% of total binding, and all binding assays were performed in hypotonic buffer to minimize possible accumulation of drug in the intravesicular space. Membranes from the parental cell line AuxB1, which does not express P-gp, displayed negligible specific binding of the radioligand (data not shown).

The relative abilities of XR9051 and Vpm to displace the equilibrium binding of [$^3$H]vinblastine to CH$^r$B30 membranes is shown in Figure 6, and data obtained using XR9051, Vpm and CsA are summarized in Table 3.

XR9051 ($EC_{50}$ = 1.40 ± 0.53 nM) was significantly more potent ($P < 0.05$) than CsA ($EC_{50}$ = 4.37 ± 0.80 nM) in displacing [$^3$H]vinblastine binding to P-gp. In contrast, Vpm was approximately 1000-fold less potent ($EC_{50}$ = 1270 ± 740 nM). Although very potent in displacing vinblastine binding, XR9051 (final fraction bound = 0.35) did not completely inhibit the binding of [$^3$H]vinblastine to P-gp in contrast to CsA and Vpm.

### DISCUSSION

This current study was undertaken to characterize a specific inhibitor of P-gp that is more potent and lacks the side-effects of some of the modulators currently undergoing clinical assessment in refractory cancers. Extensive modification of a natural product lead resulted in XR9051, which was found to be active at submicromolar concentrations in a number of assays assessing P-gp-dependent MDR.

Several assays were used to examine the inhibition of P-gp transport and the direct interaction between XR9051 and its target. Drug accumulation assays were used to demonstrate that XR9051 reversed the accumulation deficit associated with overexpression of P-gp. In the murine EMT6/AR 1.0 mammary carcinoma cell line, XR9051 was more potent than both CsA and Vpm in restoring [$^3$H]daunorubicin accumulation. XR9051 also proved highly potent in reversing the accumulation deficit of the fluorescent probes calcein-AM and rhodamine-123 in human and murine cell lines (data not shown). In contrast to the resistant cell lines, XR9051 had little effect on drug accumulation in parental cell lines, indicating that it exerted its influence through inhibition of P-gp function. Furthermore, XR9051 showed no activity in a cell line whose resistance to cytotoxic drugs is mediated by the multidrug resistance-associated protein, MRP (data not shown). Thus, XR9051 appears to be a specific inhibitor of P-gp-mediated transport. Evidence for a direct interaction between XR9051 and P-gp was implied by the photoaffinity labelling studies using the photoreactive P-gp substrate azidopine. XR9051 appeared to be less potent than CsA in this assay, which suggests that the two modulators may not interact with P-gp at exactly the same site. This interaction was confirmed by the ability of XR9051 to displace the binding of [$^3$H]vinblastine to P-gp. In this assay XR9051 was significantly more potent than CsA and Vpm.

© Cancer Research Campaign 1998

Nevertheless, XR9051 was not able to completely displace [$^3$H]vinblastine binding, possibly due to an interaction at a region distinct from the vinblastine binding site as has been proposed for 1,4-dihydropyridines (Ferry et al, 1992). Such a mechanism of action for XR9051 may explain the discrepancy between the concentrations needed to displace [$^3$H]vinblastine and those needed to reverse MDR in cell-based assays. Confirmation of this hypothesis awaits kinetic studies on drug binding.

The characterization of XR9051 was extended to assess the reversal of the MDR phenotype in a number of cell lines, where the restoration of cytotoxic drug accumulation in MDR cell lines was found to correlate with the restoration of cytotoxic-mediated cell kill. XR9051 was found to be effective in potentiating the toxicity of several clinically relevant MDR drugs in a range of murine and human P-gp-overexpressing MDR cell lines. The cell lines examined included those of leukaemic origin as well as cell lines derived from solid tumours such as breast, ovary and lung. A concentration of 0.3–0.5 μM was sufficient for full reversal of resistance. In most cases significant potentiation of activity was seen at concentrations of 0.1 μM, indicating that XR9051 is one of the more potent modulators known. An important property of an effective MDR modulator is that its activity persists in vitro for an extended period following removal from the culture medium. This feature should give the modulator a significant advantage for clinical administration. Two different transport studies demonstrated that XR9051 was particularly effective in inhibiting P-gp function for in excess of 22 h, even after removal from culture medium. This is in marked contrast to both CsA and Vpm, which are both known as P-gp substrates. This difference in properties may suggest that XR9051 is not a substrate for export by P-gp and may give further evidence that XR9051 binds to a distinct but linked site to that of cytotoxics and other P-gp substrates. Nevertheless, it should be noted that the ability of a P-gp inhibitor to retain activity over an extended period may affect the tissue distribution and pharmacokinetics of other compounds. Studies in mdr1a (–/–) knockout mice have suggested that P-gp can influence the pharmacology of drugs other than anti-cancer agents, especially in the blood–brain barrier (Schinkel et al, 1995).

In summary, XR9051 has been demonstrated to be a potent and specific inhibitor of P-gp function in vitro, with a superior duration of action to other agents tested. The compound is significantly more potent than CsA and Vpm. Furthermore, on the basis of published data, XR9051 is more potent than S9788 (Pierré et al, 1992) and VX710 (Germann et al, 1997). XR9051 is effective in restoring the sensitivity of many different tumour types to numerous MDR chemotherapeutic agents and therefore has great potential in the treatment of refractory cancers.

### ACKNOWLEDGEMENT

The authors would like to acknowledge gratefully the work of the Medicinal Chemistry Department at Xenova for synthesizing the XR9051 used in these studies.

### REFERENCES

Barrand MA, Rhodes T, Center MS and Twentyman PR (1992) Chemosensitisation and drug accumulation effects of cyclosporin A, PSC-833 and verapamil in human MDR large cell lung cancer cells expressing a 190 k membrane protein distinct from P-glycoprotein. *Eur J Cancer* **29A**: 408–415

Boesch D and Loor F (1994) Extent and persistence of P-glycoprotein inhibition in multidrug-resistant P388 cells after exposure to resistance-modifying agents. *Anticancer Drugs* **5**: 229–238

Boesch D, Gaveraux C, Jachez B, Pourtier-Manzanedo A, Bollinger P and Loor F (1991) *In vivo* circumvention of P-glycoprotein-mediated multidrug resistance of tumour cells with SDZ PSC 833. *Cancer Res* **51**: 4226–4233

Callaghan R, Berridge G, Ferry DR and Higgins CF (1997) The functional purification of P-glycoprotein is dependent on maintenance of a lipid-protein interface. *Biochim Biophys Acta* **1328**: 109–124

Dantzig AH, Shepard RL, Cao J, Law KL, Ehldardt WJ, Baughman TM, Bumol TF and Starling JJ (1996) Reversal of P-glycoprotein-mediated multidrug resistance by a potent cyclopropyldibenzosuberane modulator, LY335979. *Cancer Res* **56**: 4171–4179

De Lean A, Munson PJ, Rodbard D (1978) Simultaneous analysis of families of sigmoidal curves: applications to bioassay, radioligand assay, and physiological dose-response curves. *Am J Physiol* **235**: E97–E102

Ferry DR, Rombusch M, Goll A and Glossmann H (1984) Photoaffinity labelling of $Ca^{2+}$ channels with [$^3$H]azidopine. *FEBS Lett* **169**: 112–118

Ferry DR, Russel MA and Cullen MH (1992) P-glycoprotein possesses a 1,4-dihydropyridine selective drug acceptor site which is allosterically coupled to a vinca alkaloid selective binding site. *Biochem Biophys Res Commun* **188**: 440–445

Ford JM (1996). Experimental reversal of P-glycoprotein-mediated multidrug resistance by pharmacological chemosensitisers. *Eur J Cancer* **32A**: 991–1001

Germann UA, Shlyakhter D, Mason VS, Zelle RE, Duffy JP, Galullo V, Armistead DM, Saunders JO, Boger J and Harding MW (1997) Cellular and biochemical characterization of VX-710 as a chemosensitizer: reversal of P-glycoprotein-mediated multidrug resistance *in vitro*. *Anticancer Drugs* **8**: 125–140

Gottesman MM, Pastan I (1993) Biochemistry of multidrug resistance mediated by the multidrug transporter. *Annu Rev Biochem* **62**: 385–427

Greenberger LM, Williams SS, Georges E, Ling V and Horwitz SB (1988) Electrophoretic analysis of P-glycoproteins produced by mouse J774.2 and chinese hamster ovary multidrug resistant cells. *J Natl Cancer Inst* **80**: 506–510

Hyafil F, Vergely C, Du Vignaud P and Grand-Perret T (1993) *In vitro* and *in vivo* reversal of multidrug resitance by GF120918, an acridone carboxamide derivative. *Cancer Res* **53**: 4595–4602

Johnson RK, Chitnis MP, Embrey WM and Gregory EB (1978) *In vivo* characteristics of resistance and cross-resistance of an adriamycin-resistant subline of P388 leukaemia. *Cancer Treat Rep* **62**: 1535–1547

Kartner N, Riordan JR, and Ling V (1983) Cell surface P-glycoprotein associated with multidrug resistance in mammalian cell lines. *Science* **221**: 1285–1288

Lever JE (1977) Active amino-acid transport in plasma membrane vesicles from Simian virus 40-transformed mouse fibroblasts. Characteristics of electrochemical Na+ gradient stimulated uptake. *J Biol Chem* **252**: 1990–1997

Lum BL, Fisher GA, Brophy NA, Yahanda AM, Adler KM, Kaubisch S, Halsey J and Sikic BI (1993) Clinical trials of modulation of multidrug resistance. Pharmacokinetic and pharmacodynamic considerations. *Cancer* **72** (suppl): 3502–3514

Pierré A, Dunn TA, Kraus-Berthier L, Léonce S, Saint-Dizier D, Regnier G, Dhainaut A, Berlion M, Bizzari JP, Atassi G (1992) *In vitro* and *in vivo* circumvention of multidrug resistance by Servier 9788, a novel triazinoaminopiperidine derivative. *Invest New Drugs* **10**: 137–148

Raderer M and Scheithauer W (1993) Clinical trials of agents that reverse multidrug resistance. *Cancer* **72**: 3553–3563

Rogan AM, Hamilton TC, Young RC, Klecker RW and Ozols RF (1984) Reversal of adriamycin resistance by verapamil in human ovarian cancer. *Science* **224**: 994–996

Schinkel AH, Wagenaar E, van Deemter L, Mol CAAM, Borst P (1995) Absence of the mdr1a P-glycoprotein in mice affects tissue distribution and pharmacokinetics of dexamethasone, digoxin and cyclosporin A. *J Clin Invest* **96**: 1698–1705

Simon SM and Schindler M (1994) Cell biological mechanisms of multidrug resistance in tumors. *Proc Natl Acad Sci USA* **91**: 3497–3504

Skehan P, Storeng R, Scudiero D, Monks A, McMahon J, Vistica D, Warren JT, Bokesch H, Kenney S and Boyd MR (1990) New colorimetric cytotoxicity assay for anticancer-drug screening. *J Natl Cancer Inst* **82**: 1107–1112

Spoelstra EC, Westerhoff HV, Pinedo HM, Dekker H and Lankelma J (1994) The multidrug-resistance-reverser verapamil interferes with cellular P-glycoprotein-mediated pumping of daunorubicin as a non-competing substrate. *Eur J Biochem* **221**: 363–373

Twentyman PR, Fox NE, Wright KA and Bleehen NM (1986) Derivation and preliminary characterisation of adriamycin resistant lines of human lung cancer cells. *Br J Cancer* **53**: 529–537

Twentyman PR. Reeve JG. Koch G and Wright KA (1990) Chemosensitization by verapamil and cyclosporin A in mouse tumour cells expressing different levels of P-glycoprotein and CP22 (sorcin). *Br J Cancer* **62**: 89–95

Yang CP. Mellado W and Horwitz SB (1988) Azidopine photoaffinity labeling of multidrug resistance-associated glycoproteins. *Biochem Pharmocol* **37**: 1417–1421

Zhou D-C. Simonin G. Faussat A-M. Zittou R. Marie J-P (1997) Effect of the multidrug inhibitor GG918 on drug sensitivity of leukemic cells. *Leukemia* **11**: 1516–1522

*© Cancer Research Campaign 1998*